# Rumination as a Mediator between Childhood Trauma and Adulthood Depression/Anxiety in Non-clinical Participants

**DOI:** 10.3389/fpsyg.2017.01597

**Published:** 2017-09-25

**Authors:** Ji S. Kim, Min J. Jin, Wookyoung Jung, Sang W. Hahn, Seung-Hwan Lee

**Affiliations:** ^1^Clinical Emotion and Cognition Research Laboratory, Inje University Goyang, South Korea; ^2^Department of Psychiatry, Soonchunhyang University of College of Medicine Chunan, South Korea; ^3^Department of Psychology, Chung-Ang University Seoul, South Korea; ^4^Department of Psychiatry, Soonchunhyang University of College of Medicine Seoul, South Korea; ^5^Department of Psychiatry, Inje University, Ilsan-Paik Hospital Goyang, South Korea

**Keywords:** childhood trauma, rumination, mood, mediation, structural equation modeling

## Abstract

**Objective:** Although there is strong evidence that childhood trauma is associated with the development of depression and anxiety, relatively few studies have explored potential mediating factors for this relationship. The present study aimed to evaluate the mediating role of rumination in the link between childhood trauma and mood status such as depression, anxiety and affective lability.

**Materials and Methods:** Two hundred and seven non-clinical participants completed the Childhood Trauma Questionnaire, the Ruminative Response Scale, the Beck Depression Inventory, the State Anxiety Inventory, and the Affective Lability Scale. Structural equation modeling was used to evaluate the results.

**Results:** Our results supported that rumination is a meaningful mediator between childhood trauma and depression/anxiety in non-clinical participants. The mediation model indicated that childhood trauma and its subtypes are linked to depression and anxiety through three subtypes of rumination, thereby supporting a significant indirect relationship (Standardized coefficient [SC] = 0.56, *p* < 0.001 for the path from trauma to rumination; SC = 0.67, *p* < 0.001, from rumination to mood). The direct relationship between childhood trauma and mood symptoms was also significant in a model including rumination (SC = 0.68, *p* < 0.001). The mediation effect of rumination in the relationship between childhood trauma and mood was more predominant in female participants.

**Conclusions:** The present study found that rumination mediates the influence of childhood trauma on the development of mood symptoms in non-clinical participants. Childhood trauma appears to be a critical determinant for developing symptoms of depression and anxiety.

## Introduction

Childhood trauma in terms of abuse and neglect has been quite common not only in pathological conditions but also in community population (Kilpatrick and Saunders, 2000). Moreover, childhood trauma has been regarded as an origin for various psychopathologies (Hetzel and McCanne, 2005; Nanni et al., 2012). In particular, childhood trauma has been associated with the development of depression and anxiety (Chapman et al., 2004; Sachs-Ericsson et al., 2006; Lok et al., 2013). Recently, it was revealed that the affective lability or bipolar disorder as well as the unipolar depression have also been associated with childhood traumatic experiences (Aas et al., 2014; Noto et al., 2015; Marwaha et al., 2016).

Although relatively few studies have explored potential mediating mechanisms of the relationship between childhood trauma and mood status, some mediating candidates have been reported. Maladaptive cognition and negative cognitive styles have been suggested to mediate the link between childhood maltreatment and depression (Gibb et al., 2001; Kaysen et al., 2005; Hankin, 2006). Recently, dysfunctional emotional regulation has been suggested as an underlying mediator of the association between childhood trauma and depression (Hopfinger et al., 2016). In addition, rumination has been another plausible candidate in this relationship (Raes and Hermans, 2008). Rumination is defined as the tendency to constantly focus on negative mood and on the possible causes and implications of depressed feelings (Nolen-Hoeksema, 1991). Rumination has been regarded as one of the underlying mechanisms for developing and maintaining depression (Nolen-Hoeksema et al., 2008). Rumination affects the development of depression by reducing the specificity of autobiographical memory (Raes et al., 2006). That is, compared with controls, people with depression respond relatively more often with overgeneral or categoric memories that summarize across categories of similar events (Raes et al., 2006). Therefore, rumination might mediate developing of depression by extortion and re-production of the childhood traumatic experiences. In this context, previous studies have reported that rumination played a mediating role between emotional abuse and depression (Spasojevic and Alloy, 2001; Raes and Hermans, 2008). Although other subtypes of childhood abuse could also mediate the development of depression (Al Odhayani et al., 2013), the role of rumination as a mediator for depression remains unclarified in other types of childhood trauma.

Most previous studies applied two-factor model for rumination including brooding and reflection (Nolen-Hoeksema et al., 2008; Raes and Hermans, 2008). However, three-factor models for rumination showed better fit for the Korean population compared with two–factor models (Kim et al., 2013). Furthermore, most of the previous research has focused on depression, disregarding a variety of other mood conditions such as anxiety or mood lability. This is the first study that aims to evaluate the mediating role of three subtypes of rumination including self-reproach, contemplation, and depressive rumination, in the relationship between five subtypes of childhood trauma and various mood-related conditions, namely depression, anxiety and affective lability, using structural equation modeling in the general population. This study considered depression, anxiety and affective lability as mood-related conditions for convenience of readers. Moreover, previous studies have reported that female participants tend to use more ruminative response than males (Nolen-Hoeksema et al., 1999; Johnson and Whisman, 2013). Therefore, the study additionally accounted for sex and investigated its influence on the mediating role of rumination in the relationship between childhood trauma and mood-related condition.

The present study hypothesized that subtypes of rumination mediate the effect of childhood trauma on adult's mood-related conditions such as depression, anxiety, and affective lability. Furthermore, the study hypothesized that this mediating role is predominantly evident in female participants.

## Materials and methods

### Participants

The study was performed on 207 non-clinical volunteers (84 men and 123 women) with a mean age of 27.86 ± 6.36 (years). They were recruited from the local community through local newspapers and posters. Participants with any history of neurological or other mental diseases were excluded from the study through the initial screening interviews. After an explanation of this study, all participants signed an informed consent that was approved by the Institutional Review Board at Inje University Ilsan Paik Hospital prior to their participation (IRB no. 2015-07-026-001).

### Psychological measures

Participants completed the Korean version of Childhood Trauma Questionnaire-Short Form (K-CTQ) (Bernstein and Fink, 1998) to assess their childhood traumatic experiences. The Childhood Trauma Questionnaire-Short Form (CTQ-SF) is a 28-item self-report inventory developed to measure five subtypes of abuse or neglect in childhood or adolescence: sexual abuse, physical abuse, emotional abuse, physical neglect, and emotional neglect (Bernstein et al., 2003). Each Item has a five-point, Likert-type answer format ranging from “never true” (score = 1) to “very often true” (score = 5). Good to excellent internal consistency for each subscale except physical neglect has been obtained from community populations (Scher et al., 2001). The K-CTQ showed adequate reliability and validity (Kim et al., 2011). Its coefficient alpha was 0.90 in this study. The coefficients of each subtype of K-CTQ were 0.92 (emotional neglect), 0.78 (physical abuse), 0.78 (sexual abuse), 0.77 (emotional abuse) and 0.52 (physical neglect) in this study.

For the evaluation of rumination, the Ruminative Response Scale (RRS) was conducted. RRS is a validated scale composed of 22 items for measuring the ruminative responses to depressed moods (Nolen-Hoeksema, 1991; Kim et al., 2010). Each item is rated on a 4-point scale (“almost never” to “almost always”). The Korean version of RRS (K-RRS) has good internal consistency in community samples (Kim et al., 2010). The K-RRS consists of three subscales and each subscale has an adequate internal consistency (Cronbach's alpha = 0.80 for self-reproach, 0.79 for contemplation, 0.86 for depressive rumination; Kim et al., 2010). The three factors of rumination were self-reproach, contemplation, and depressive rumination. The self-reproach means blaming by one's own conscience and the contemplation is defined by profound thinking about depressed mood or personality. The depressive rumination is the focused attention on the symptoms of one's depressive mood (Kim et al., 2010). Previous exploratory and confirmatory factor analysis for K-RRS indicated that the above-mentioned three-subscale model was more suitable for both Korean non-clinical and clinical populations (Kim et al., 2010, 2013) than two-subscale (brooding/reflection) model (Raes and Hermans, 2008). The K-RRS coefficient alpha in this study was 0.92. The coefficients of each subtype of K-RRS were 0.85 (self-reproach), 0.87 (contemplation) and 0.81 (depressive rumination) in this study.

For the assessment of mood variables, the State Anxiety Inventory (SAI), Short form of Affective Lability Scale (ALS-SF), and Beck Depression Inventory (BDI) were used. The SAI is a subscale of State and Trait Anxiety Inventory, and is comprised of 20 items (Kim and Shin, 1978; Spielberger, 1983) that measure an immediate emotional status caused by concern or tension, which are changeable depending on the patient's current status. SAI consists of a four-point scale and each item is rated from “not at all” to “very much so” (Hahn et al., 1996). The Korean version of SAI showed adequate reliability and validity (Hahn et al., 1996). Its coefficient alpha was 0.92 in this study.

The ALS-SF was utilized to estimate instability of mood (Harvey et al., 1989; Aas et al., 2015). It is consist of three subscales such as anxiety/depression scale, depression/elation scale, and an anger scale. It is comprised of 18 items and assessed with a 4-point likert scale. Its coefficient alpha was 0.93 in this study.

The BDI is a validated scale composed of 21 items for measuring the severity of depressive symptoms (Beck et al., 1961; Rhee et al., 1995). BDI showed good internal consistency and validity (Song et al., 2012). Its coefficient alpha was 0.87 in this study.

### Statistical analysis

Descriptive statistics for demographic and psychological characteristics of participants were performed using SPSS 21 (SPSS, Inc., Chicago, IL, USA). The significant level was set at *p* < 0.05 (two-tailed).

Normality was tested for each variable before further analysis. The skewness over 2.0 and kurtosis over 7.0 are considered to be a moderately non-normal distribution (Curran et al., 1996). All variables in our results were within the range of normal distribution except for sexual abuse (skewness was 3.51 and Kurtosis was 14.59) and emotional abuse (skewness was 2.22).

Independent *t*-test was used to compare the scores of psychological data between the male and female group. The psychological data were normally distributed in both the male and female group. Pearson's correlation analysis was performed with psychological measures that showed a normal distribution.

Structural equation modeling (SEM) was used to assess hypothesized relationships between childhood traumatic experiences, rumination, and mood status in a non-clinical population. SEM is defined as the combination of confirmatory factor analysis and multiple regressions to determine relationships between variables (Sergi et al., 2006). Regarding the factor-analytic properties of SEM, *unobserved variables* are estimated by factor analyses of data from theoretically related measures, *observed variables*. Factor loadings are used to specify the association between *unobserved variables* and *observed variables* (Kline, 2011). The relations between the *unobserved variables* were analyzed by regression analysis. Additionally, the study conducted the bootstrap procedure to estimate the size of the indirect effects (*n* = 5,000 resamples) (Preacher and Hayes, 2004). The study also used the bias-corrected bootstrap sampling for the maximum likelihood estimation to minimize potential bias to model fits (Preacher and Hayes, 2004). The confidence interval (CI) for the indirect effect was a BCa bootstrapped CI, and the significance of the point estimate (*p* < 0.05) was determined by the absence of zero within the CI (Preacher and Hayes, 2004; Hopfinger et al., 2016).

As the data were non-normal in distribution and we used the Mardia's test (Mardia, 1970). The result of Mardia's test of multivariate normality was highly significant (*z* = 27.04, *p* < 0.001). Clearly, these data are not multivariate normal. However, the model in this study was examined via SEM using the maximum- likelihood method that applies the bootstrapping approach, which does not require a distributional assumption and estimates the standard errors for parameter estimates using the bootstrap algorithm of Efron (1982). Previous researchers suggested that the maximum likelihood method with large sample sizes could be used if the data were non-normal in distribution (Hu et al., 1992; Yuan and Bentler, 1999). Additionally, the parameter estimation could be reliable when the used maximum likelihood method although the multivariate normality assumption was not satisfied (Hair et al., 2010). Therefore this study the used maximum likelihood method with bootstrapping as parameter estimation owing to acceptable skewness and kurtosis (Curran et al., 1996).

The present model consisted of three unobserved variables: childhood trauma, rumination, and mood. The variable called *childhood trauma* has five observed variables: emotional abuse, emotional neglect, physical abuse, physical neglect, and sexual abuse. The second unobserved variable is *rumination* and has three observed variables: self-reproach, contemplation, and depressive rumination. The third unobserved variable, *mood*, has three observed variables: depression, anxiety, and affective lability. Two models, a basic model and a mediation model, were evaluated. The basic model verified the direct relationship between childhood trauma and mood. The mediation model evaluated both direct and indirect relationships between childhood trauma and mood through mediation of rumination.

Overall model fit was evaluated using the following criteria. First, the present study calculated the ratio of *x*^2^ to degrees of freedom that should be less than three as an acceptable data-model fit (Fino et al., 2014). In addition, the study also used the Comparative Fit Index (CFI) and the Root Mean Square Error of Approximation (RMSEA). Indicators of adequate-fitting model are evidenced by an CFI greater than 0.95 and an RMSEA less than 0.08 (MacCallum et al., 1996).

Additionally, to test the significance of a mediation effect, this study conducted the Sobel test (Sobel, 1982), which is an interactive method for testing whether a mediator variable significantly carries the influence of an independent variable to a dependent variable.

The study examined the hypothesized relations in our model using AMOS 21 for SEM (SPSS Inc.) (Arbuckle, 1994). Statistical analyses were performed using SPSS 21 (SPSS, Inc., Chicago, IL, USA).

## Results

The means and standard deviations (SD) of the psychological characteristics related to the observed variables are presented in Table 1. The correlation analyses of the psychological measures are displayed in Table 2. Correlation analyses demonstrated that associations between observed variables were in the expected directions.

**Table 1:** Psychological characteristics of the participants.

**Psychological measures**	**Total (*N* = 207)**	**Men (*N* = 84)**	**Women (*N* = 123)**	
	**Mean** ±***SD***			***p*-value**
Childhood trauma questionnaire (CTQ)	43.29 ± 12.20	41.77 ± 11.35	44.33 ± 12.69	0.14
Emotional abuse	6.42 ± 2.57	5.93 ± 2.01	6.76 ± 2.85	0.02
Emotional neglect	17.01 ± 6.73	15.86 ± 6.49	17.80 ± 6.81	0.04
Physical abuse	7.24 ± 2.99	7.37 ± 2.90	7.15 ± 3.06	0.61
Physical neglect	6.69 ± 2.32	6.96 ± 2.55	6.50 ± 2.15	0.16
Sexual abuse	5.93 ± 2.15	5.65 ± 1.52	6.11 ± 2.49	0.13
Ruminative response scale	33.25 ± 9.04	32.24 ± 8.26	33.93 ± 9.51	0.05
Self-reproach	11.61 ± 3.81	11.33 ± 4.07	11.80 ± 3.63	0.39
Contemplation	11.15 ± 3.94	10.90 ± 3.91	11.80 ± 3.97	0.46
Depressive rumination	10.66 ± 3.18	10.43 ± 2.98	10.82 ± 3.31	0.38
State anxiety inventory (SAI)	36.84 ± 8.30	35.69 ± 7.99	37.63 ± 8.45	0.09
Beck Depression Inventory (BDI)	8.26 ± 5.91	7.30 ± 5.67	8.91 ± 6.01	0.05
Affective lability scale (ALS)	16.78 ± 9.89	13.48 ± 9.38	19.03 ± 9.63	<0.001

*SD, standard deviation*.

**Table 2:** Correlations of psychological measures in the participants.

	***r***										
**Measures**	**1**	**2**	**3**	**4**	**5**	**6**	**7**	**8**	**9**	**10**	**11**
**CHILDHOOD TRAUMA**											
1. Emotional abuse^a^	-										
2. Emotional neglect	0.46^**^	-									
3. Physical abuse	0.39^**^	0.34^**^	-								
4. Physical neglect	0.24^**^	0.29^**^	0.14	-							
5. Sexual abuse^a^	0.35^**^	0.30^**^	0.37^**^	0.22^**^	-						
**RUMINATION**											
6. Self-reproach	0.45^**^	0.34^**^	0.38^**^	0.21^**^	0.13	-					
7. Contemplation	0.40^**^	0.32^**^	0.24^**^	0.15	0.16^*^	0.47^**^	-				
8. Depressive rumination	0.45^**^	0.40^**^	0.59^**^	0.17^*^	0.14	0.63^**^	0.61^**^	-			
**MOOD**											
9. State Anxiety	0.29^**^	0.37^**^	0.29^**^	0.25^**^	0.09	0.50^**^	0.30^**^	0.41^**^	-		
10. Beck Depression	0.43^**^	0.44^**^	0.26^**^	0.17^*^	0.10	0.60^**^	0.37^**^	0.59^**^	0.54^**^	-	
11. Affective Lability	0.38^**^	0.30^**^	0.23^**^	0.13	0.24^**^	0.45^**^	0.40^**^	0.51^**^	0.46^**^	0.47^**^	-

* *p < 0.05*,

** *p < 0.01*.

a *Spearman correlation analysis was performed*.

**Figure 1:**
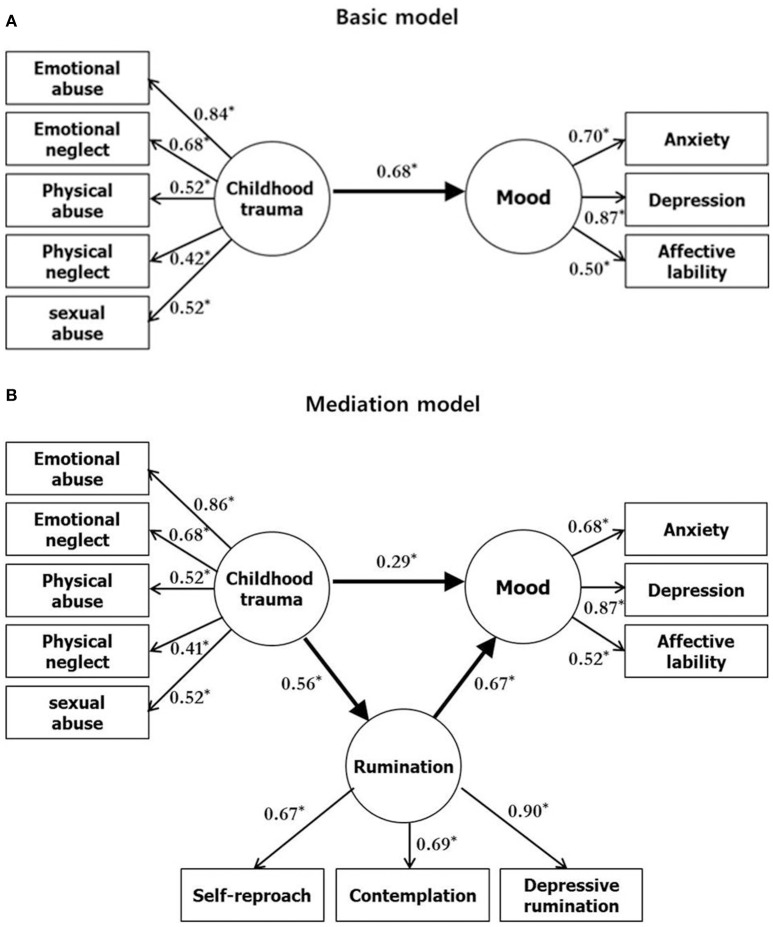
Basic model of the relationship between childhood trauma and mood **(A)**, and mediation model showing rumination as a mediator of the relationship **(B)**. Rectangles represent observed variables. Circles represent latent variables. Numbers on single-headed arrows indicate standardized regression weights. ^*^p < 0.05, multiple regression analysis.

On the basis of our previous hypothesis, the basic and mediation models were estimated with the maximum likelihood method. The basic model showed a direct relationship between childhood trauma and mood (Figure 1A). Therefore, the basic model is satisfied with an adequate fit for the data, and produced fit indices as follows: *x*^2^/df = 1.95; CFI = 0.96; RMSEA = 0.06. As the authors expected, childhood trauma, indicated by the subtypes of childhood trauma questionnaire, predicted mood status indicated by BDI, SAI, and ALS in the basic model (Standardized coefficient = 0.68, *p* < 0.001).

The mediation model showed the strength of a direct relationship between childhood trauma and mood status, as well as an indirect relationship mediated by rumination (Figure 1B). The model produced adequate fit indices as follows: *x*^2^/df = 2.18; CFI = 0.94; RMSEA = 0.07. Similarly to the basic model, all observed variables included in the mediation model had moderate to high loadings in their respective unobserved variables, except for *physical neglect*. Rumination and its three observed variables (subtypes of rumination) were predicted by childhood trauma (Standardized coefficient = 0.56, *p* < 0.001) and were predictive of mood status (Standardized coefficient = 0.67, *p* < 0.001). Rumination mediated the relationship between the predictor and outcome variables, as indicated by the significant indirect path between K-CTQ and mood (mood: *p* = 0.006, ALS: *p* = 0.008, SAI: *p* = 0.003, BDI: *p* = 0.004). The Sobel test, which was conducted in order to evaluate the significance of the mediation effect, resulted in a high 4.19, suggesting that the mediating effect of rumination was significant.

The basic model (Figure 1A) showed a significant direct effect (*p* = 0.01, *b* = 0.68) while the mediation model (Figure 1B) showed both significant direct (*p* = 0.01, *b* = 0.29, CI [0.003, 0.49]) and indirect (*p* = 0.01, *b* = 0.38, CI [0.25, 0.56]) effects between childhood trauma and mood through the mediation of rumination. In the model (Figure 1B), the total effect was 0.67. The direct effect was 0.29 and indirect effect was 0.38.

**Figure 2:**
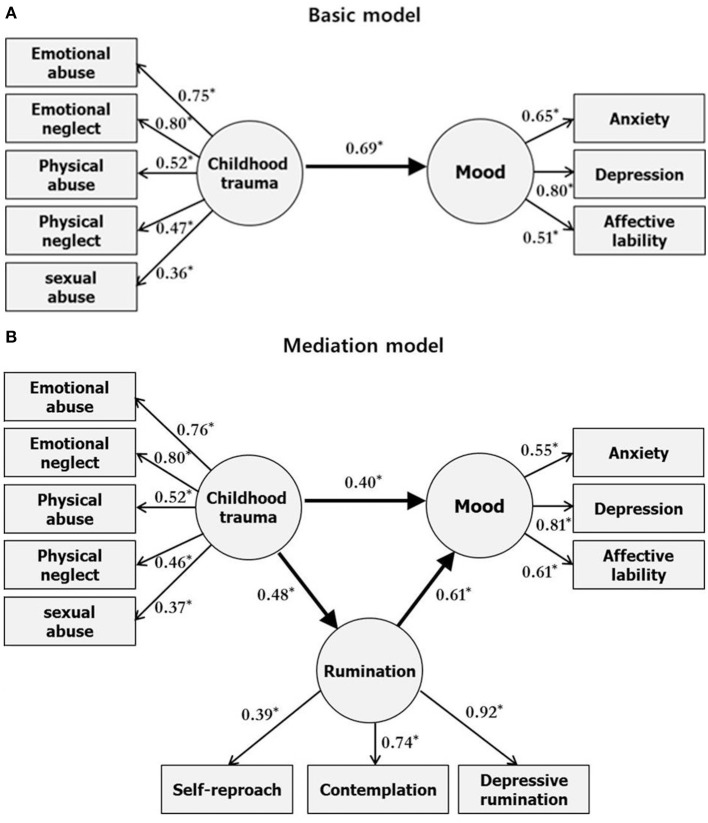
Basic model of the relationship between childhood trauma and mood **(A)**, and mediation model showing rumination as a mediator of the relationship **(B)** in male participants. Rectangles represent observed variables. Circles represent latent variables. Numbers on single-headed arrows indicate standardized regression weights. ^*^p < 0.05, multiple regression analysis.

**Figure 3:**
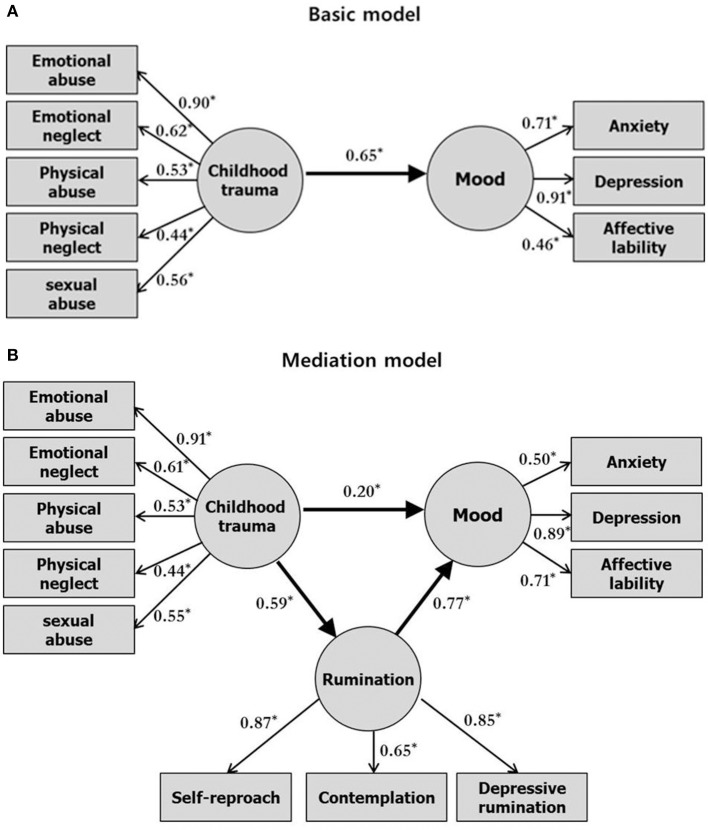
Basic model of the relationship between childhood trauma and mood **(A)**, and mediation model showing rumination as a mediator of the relationship **(B)** in female participants. Rectangles represent observed variables. Circles represent latent variables. Numbers on single-headed arrows indicate standardized regression weights. ^*^p < 0.05, multiple regression analysis.

The study further conducted the analysis with sex as a factor. The analysis conducted only on male participants is presented in Figure 2. The analysis conducted only on female participants is presented in Figure 3. The mediation model in male participants produced very good fit indices as follows: *x*^2^ /df = 1.30; CFI = 0.95; RMSEA = 0.06. The mediation model in female participants produced adequate fit indices as follows: *x*^2^/df = 1.90; CFI = 0.93; RMSEA = 0.08. In male participants, the basic model (Figure 2A) showed a significant direct effect (*p* = 0.04) and the mediation model (Figure 2B) showed a significant direct (*p* = 0.04, b = 0.40, CI [0.06, 0.75]) and indirect (*p* < 0.001, *b* = 0.29, CI [0.12, 0.58]) effect between childhood trauma and mood through the mediation of rumination. In female participants, the basic model (Figure 3A) showed a significant direct effect (*p* = 0.04) and the mediation model (Figure 3B) showed a significant direct (*p* = 0.04, *b* = 0.20, CI [0.02, 0.40]) and indirect (*p* < 0.001, *b* = 0.45, CI [0.28, 0.69]) effect between childhood trauma and mood through the mediation of rumination. In the analysis conducted by taking the sex variable into account, the standardized indirect effect size was larger in female participants than in male participants (female = 0.45, male = 0.29).

## Discussion

The present study aimed to clarify whether rumination would mediate the path from childhood trauma to a mood-related condition in a non-clinical population. As expected, rumination acted as a mediator between various types of childhood trauma and mood-related condition. Additionally, this mediation effect has been observed more predominantly in female participants.

### Does childhood trauma induce depression, anxiety and affective lability?

The model showed a direct relationship between childhood trauma and mood. All observed variables included in this model had moderate to high loadings on their respective unobserved variables, except for the physical neglect variable of the childhood trauma questionnaire. The physical neglect variable showed lower factor loading in a confirmatory factor analysis of childhood trauma questionnaire (Scher et al., 2001). Previous studies reported that the subscale physical neglect was loaded in different factors (Villano et al., 2004; Gerdner and Allgulander, 2009; Klinitzke et al., 2012). Researchers insisted that this problem may be due to the poor differentiation between physical neglect and emotional neglect or because these two separate factors are conceptually intermingled in the construct of physical neglect (Kim et al., 2011; Grassi-Oliveira et al., 2014).

Individuals with a history of childhood trauma often struggle with various symptom complexes including depression, anxiety and lability (Gillespie and Nemeroff, 2005; Aas et al., 2014). A lot of studies suggest that disruption of stress-related neural systems, which might be induced by early childhood trauma, plays a critical role in the development of depression and anxiety (Heim et al., 2004). In this process, the role of corticotropin-releasing factor (CRF) is preeminent (Gillespie and Nemeroff, 2005). A previous study observed that childhood sexual abuse was associated with an increased sensitivity to the depressogenic effects of stressful life events (Kendler et al., 2004). Moreover, genetic polymorphism and brain structural findings suggest that depression or anxiety related to early life stress might be a distinct form of depression (Hasler et al., 2004; Kim and Lee, 2016). Among the several risk factors related to affective lability, the childhood trauma was specially regarded to relevant one. Moreover, a recently reported study suggested that higher affective lability leading from childhood trauma even affected poor clinical outcomes of mood disorders such as suicidal attempts (Aas et al., 2017). Our study was in line with previous studies suggesting that childhood trauma can directly predict adulthood mood status.

### Does rumination reliably mediate the effect of childhood trauma on depression, anxiety and affective lability?

Besides these apparent associations between early traumatic experiences and later life mood problems, data on the causal mechanisms of this pathway are scarce. Our results showed that rumination is an important factor in the pathway between childhood trauma and mood-related condition later in life. Previously, it has been revealed that childhood traumatic experiences are related to the development of depression mediated by rumination (Spasojevic and Alloy, 2001; Raes and Hermans, 2008). Importantly, our results extend the previous findings by evaluating other stressful symptoms such as anxiety (Nolen-Hoeksema, 2000; Marcus et al., 2008), and mood lability (Marcus et al., 2008). Moreover, our results show that the indirect model of rumination has a significant mediating effect for mood symptoms. It suggests that rumination could be an important mediator underlying the development of various mood problems including affective lability, anxiety, and depression caused by early traumatic experiences. Furthermore, it gives us an important clinical implication that the ruminative cognitive styles should be targets for treatment in childhood trauma victims to prevent mood disorders.

### Why the rumination is important as a mediator?

Our study revealed that all subtypes of ruminative response such as self-reproach, contemplation, and depressive rumination could be associated with childhood trauma. This is the first demonstration that showed possibilities for the mediating role of subtypes of rumination in the relationship between various subtypes of childhood trauma and development of depression, anxiety and affective lability. A previous study has revealed that emotional abuse is associated with the development of emotional dysregulation with rumination as a mediator (Raes and Hermans, 2008). Traumas that have not been fully discussed and ventilated are linked to increased ruminations about that traumatic event (Pennebaker and Susman, 1988). Generally, childhood trauma is not easily discussed and is suppressed, so that it might lead to a ruminative cognitive style. Previous studies have demonstrated that childhood maltreatment might be one developmental antecedent of rumination (Gold and Wegner, 1995; Nolen-Hoeksema, 2004). Rumination after stress predicted non-habituation of hypothalamic–pituitary–adrenal (HPA) axis responses (Gianferante et al., 2014). Increased rumination after experiencing a novel stressor was predictive of increased cortisol reactivity to second-time stress. These evidences suggest that rumination is one possible mechanism mediating maladaptive stress response patterns, and it might offer a pathway to lead to negative health outcomes such as depression (Gianferante et al., 2014). Unexpectedly, the sexual abuse subtype was not associated with rumination and depression/anxiety in our correlational analysis. It might have caused the characteristics of study population or data distribution (skewed). Despite of skewed distribution of sexual abuse, the present study chose “sexual abuse” as a factor to confirm the role of the development of mood-related symptoms regarding the importance of sexual abuse as a subtype of the CTQ. Further studies in general population with less skewed distributed data are needed to clarify the role of sexual abuse.

The present study showed the mediating role for rumination including each subtype from childhood trauma to various mood related conditions. Depressed mood has been triggered by life events that were associated with self-reproach (Maj and Sartorius, 2002). The self-reproach is closely associated with depressed mood and high distress (Zahn et al., 2015). The contemplation, another subtype of rumination, was associated with levels of anxiety symptoms (Dozois et al., 2004). Moreover, higher Contemplation was also related to significantly greater recognition of the negative consequences of worry (Dozois et al., 2004).

The focused attention on the symptoms of one's mood was associated with the mood conditions in bipolar disorder (Gruber et al., 2011).

### Possible female dominance in the role of rumination

In the present study, the mediation effect of rumination was more predominant in females compared with male participants. In male participants, the direct effect in path model was much larger than that of female (0.40 vs. 0.20). Moreover, the moderating effect of sex in the path model was different in our additional analysis regarding to sex (*p* = 0.11 for the limitation model of moderating effect analysis for sex). It means that the influence of sex on the mediating role of rumination is different in the relationship between childhood trauma and mood status. Women have a higher tendency to ruminate on their depressive symptoms and distress than do men (Nolen-Hoeksema, 2012; Johnson and Whisman, 2013). Our results showed that, in women, childhood traumatic experiences could result in more ruminative cognitive responses and cause more distress and frequent development of mood problems compared with men. Indeed, women are twice as likely as men to develop mood instability (Leach et al., 2008). Our results suggest that female victims of childhood trauma are highly prone to mental health problems and that they require clinical interventions to their ruminative cognitive styles in order to prevent future mood problems. Our results should be interpreted with caution because there was also a significant indirect effect of rumination in the relationship between childhood trauma and mood-related conditions in male cases and our larger sample sizes of female participants might affect the size of indirect effect. However, in the mediation model, the direct effect in male participants was larger than that of female participants. Regarding the proportion of direct/indirect effect in both sex groups, the female dominance in the role of rumination is plausible.

Cognitive behavioral interventions to reduce ruminative responses may help to prevent mood disturbances in individuals who have experienced childhood trauma.

There are some caveats in this study. First, subjects of the present study were non-clinical volunteers with various severity range of childhood trauma. Our results should be replicated in clinical subjects. Secondly, there was relatively small sample size for analysis of sex differences in the mediating role of rumination. Moreover, unequal sample sizes in each sex group might affect the results and limit interpretation of our results. A larger number of participants is a prerequisite to confirm our evidence. Thirdly, childhood trauma history was assessed retrospectively by self-report measures. However, regarding the stability and validity of retrospective self-reports of CTQ (Paivio, 2001), the chosen measure was suitable for our study. Additionally, our mediation model was cross-sectional in nature, which limits the causal relations among the assessed variables. Although our results suggest that rumination is a crucial mediator of the childhood trauma on various mood-related conditions, longitudinal prospective studies are needed to rule out other possible alternative mediators in the path model. Lastly, the present study did not use a structured clinical interview for screening possible psychiatric illness.

## Conclusions

To our knowledge, this is the first study that elucidates a comprehensive model from childhood trauma to depression, anxiety and affective lability with mediating factors of rumination including each subtype. Our data showed that childhood trauma caused mood changes, which then induced anxiety, depression, and affective lability through direct and indirect paths. Additionally, female subjects showed a higher mediating effect of ruminative cognitive styles to the path between childhood trauma and above mood-related condition. Our results present an insight on intervention for ruminative cognitive styles to prevent of development of mood problems in individuals with childhood trauma.

## Author contributions

JK is the first author. JK analyzed the data and wrote the paper. SL and SH designed the study and wrote the paper. WJ and MJ collected the data. SH reviewed and revised the paper. SL and SH are co-corresponding author and equally contributed to this study.

### Conflict of interest statement

The authors declare that the research was conducted in the absence of any commercial or financial relationships that could be construed as a potential conflict of interest.

## References

[B1] Aas M. Aminoff S. R. Vik Lagerberg T. Etain B. Agartz I. Andreassen O. A.. (2014). Affective lability in patients with bipolar disorders is associated with high levels of childhood trauma. Psychiatry Res. 218, 252–255. 10.1016/j.psychres.2014.03.046 24803185

[B2] Aas M. Henry C. Bellivier F. Lajnef M. Gard S. Kahn J. P.. (2017). Affective lability mediates the association between childhood trauma and suicide attempts, mixed episodes and co-morbid anxiety disorders in bipolar disorders. Psychol. Med. 47, 902–912. 10.1017/S0033291716003081 27894372

[B3] Aas M. Pedersen G. Henry C. Bjella T. Bellivier F. Leboyer M.. (2015). Psychometric properties of the affective lability scale (54 and 18-item version) in patients with bipolar disorder, first-degree relatives, and healthy controls. J. Affect. Disord. 172, 375–380. 10.1016/j.jad.2014.10.028 25451440

[B4] Al Odhayani A. Watson W. J. Watson L. (2013). Behavioural consequences of child abuse. Can. Fam. Physician 59, 831–836. 23946022 PMC3743691

[B5] Arbuckle J. L. (1994). AMOS-Analysis of moment strumctures. Psychometrika 59, 135–137. 10.1007/BF02294272

[B6] Beck A. T. Ward C. H. Mendelson M. Mock J. Erbaugh J. (1961). An inventory for measuring depression. Arch. Gen. Psychiatry 4, 561–571. 10.1001/archpsyc.1961.01710120031004 13688369

[B7] Bernstein D. P. Fink L. (1998). Childhood Trauma Questionnaire: a Retrospective Self-report Manual. San Antonio, TX: The Psychological Corporation.

[B8] Bernstein D. P. Stein J. A. Newcomb M. D. Walker E. Pogge D. Ahluvalia T.. (2003). Development and validation of a brief screening version of the childhood trauma questionnaire. Child Abuse Negl. 27, 169–190. 10.1016/S0145-2134(02)00541-0 12615092

[B9] Chapman D. P. Whitfield C. L. Felitti V. J. Dube S. R. Edwards V. J. Anda R. F. (2004). Adverse childhood experiences and the risk of depressive disorders in adulthood. J. Affect. Disord. 82, 217–225. 10.1016/j.jad.2003.12.013 15488250

[B10] Curran P. J. West S. G. Finch J. F. (1996). The robustness of test statistics to nonnormality and specification error in confirmatory factor analysis. Psychol. Methods 1:16 10.1037/1082-989X.1.1.16

[B11] Dozois D. J. Westra H. A. Collins K. A. Fung T. S. Garry J. K. (2004). Stages of change in anxiety: psychometric properties of the University of Rhode Island Change Assessment (URICA) scale. Behav. Res. Ther. 42, 711–729. 10.1016/S0005-7967(03)00193-1 15081886

[B12] Efron B. (1982). The Jackknife, the Bootstrap and Other Resampling Plans. Philadelphia, CA: SIAM

[B13] Fino E. Melogno S. Iliceto P. D'Aliesio S. Pinto M. A. Candilera G.. (2014). Executive functions, impulsivity, and inhibitory control in adolescents: a structural equation model. Adv. Cogn. Psychol. 10, 32–38. 10.5709/acp-0154-5 25157298 PMC4118776

[B14] Gerdner A. Allgulander C. (2009). Psychometric properties of the swedish version of the childhood trauma questionnaire-short form (CTQ-SF). Nord. J. Psychiatry 63, 160–170. 10.1080/08039480802514366 19021077

[B15] Gianferante D. Thoma M. V. Hanlin L. Chen X. Breines J. G. Zoccola P. M.. (2014). Post-stress rumination predicts HPA axis responses to repeated acute stress. Psychoneuroendocrinology 49, 244–252. 10.1016/j.psyneuen.2014.07.021 25127082 PMC4165793

[B16] Gibb B. E. Alloy L. B. Abramson L. Y. Rose D. T. Whitehouse W. G. Donovan P. (2001). History of childhood maltreatment, negative cognitive styles, and episodes of depression in adulthood. Cogn. Ther. Res. 25, 425–446. 10.1023/A:1005586519986

[B17] Gillespie C. F. Nemeroff C. B. (2005). EARLY LIFE STRESS AND DEPRESSION Childhood trauma may lead to neurobiologically unique mood disorders. Curr. Psychiatr. 4, 14–30.

[B18] Gold D. B. Wegner D. M. (1995). Origins of ruminative thought: trauma, incompleteness, nondisclosure, and supression. J. Appl. Soc. Psychol. 25, 1245–1261. 10.1111/j.1559-1816.1995.tb02617.x

[B19] Grassi-Oliveira R. Cogo-Moreira H. Salum G. A. Brietzke E. Viola T. W. Manfro G. G.. (2014). Childhood Trauma Questionnaire (CTQ) in Brazilian samples of different age groups: findings from confirmatory factor analysis. PLoS ONE 9:e87118. 10.1371/journal.pone.0087118 24475237 PMC3903618

[B20] Gruber J. Eidelman P. Johnson S. L. Smith B. Harvey A. G. (2011). Hooked on a feeling: rumination about positive and negative emotion in inter-episode bipolar disorder. J. Abnorm. Psychol. 120, 956–961. 10.1037/a0023667 21553935 PMC3409091

[B21] Hahn D. W. Lee C. H. Chon K. K. (1996). Korean adaptation of Spielberger's STAI (K-STAI). Korean J. Health Psychol. 1, 1–14.

[B22] Hair J. F. Black W. C. Babin B. J. Anderson R. E. (2010). Multivariate Data Analysis 7th Edn. Prentice Hall: Englewood Cliffs.

[B23] Hankin B. L. (2006). Childhood maltreatment and psychopathology: prospective tests of attachment, cognitive vulnerability, and stressas mediating processes. Cogn. Ther. Res. 29, 645–671. 10.1007/s10608-005-9631-z

[B24] Harvey P. D. Greenberg B. R. Serper M. R. (1989). The affective lability scales: development, reliability, and validity. J. Clin. Psychol. 45, 786–793. 10.1002/1097-4679(198909)45:5<;786::AID-JCLP2270450515>3.0.CO;2-P 2808736

[B25] Hasler G. Drevets W. C. Manji H. K. Charney D. S. (2004). Discovering endophenotypes for major depression. Neuropsychopharmacology 29, 1765–1781. 10.1038/sj.npp.1300506 15213704

[B26] Heim C. Plotsky P. M. Nemeroff C. B. (2004). Importance of studying the contributions of early adverse experience to neurobiological findings in depression. Neuropsychopharmacology 29, 641–648. 10.1038/sj.npp.1300397 15034558

[B27] Hetzel M. D. McCanne T. R. (2005). The roles of peritraumatic dissociation, child physical abuse, and child sexual abuse in the development of posttraumatic stress disorder and adult victimization. Child Abuse Negl. 29, 915–930. 10.1016/j.chiabu.2004.11.008 16125234

[B28] Hopfinger L. Berking M. Bockting C. L. Ebert D. D. (2016). Emotion regulation mediates the effect of childhood trauma on depression. J. Affect. Disord. 198, 189–197. 10.1016/j.jad.2016.03.050 27018937

[B29] Hu L. T. Bentler P. M. Kano Y. (1992). Can test statistics in covariance structure analysis be trusted? Psychol. Bull. 112, 351–362. 10.1037/0033-2909.112.2.351 1454899

[B30] Johnson D. P. Whisman M. A. (2013). Gender differences in rumination: a meta-analysis. Pers. Individ. Dif. 55, 367–374. 10.1016/j.paid.2013.03.019 24089583 PMC3786159

[B31] Kaysen D. Scher C. D. Mastnak J. Resick P. (2005). Cognitive mediation of childhood maltreatment and adult depression in recent crime victims. Behav. Ther. 36, 235–244. 10.1016/S0005-7894(05)80072-3 16467922 PMC1351207

[B32] Kendler K. S. Kuhn J. W. Prescott C. A. (2004). Childhood sexual abuse, stressful life events and risk for major depression in women. Psychol. Med. 34, 1475–1482. 10.1017/S003329170400265X 15724878

[B33] Kilpatrick D. G. Saunders B. E. (2000). “Prevalence and Consequences of Child Victimization. Results from the National Survey of Adolescents, Final Report.” (U.S. Department ofJustice).

[B34] Kim D. Park S. C. Yang H. Oh D. H. (2011). Reliability and validity of the korean version of the childhood trauma questionnaire-short form for psychiatric outpatients. Psychiatry Invest. 8, 305–311. 10.4306/pi.2011.8.4.305 22216039 PMC3246137

[B35] Kim J. S. Lee S. H. (2016). Influence of interactions between genes and childhood trauma on refractoriness in psychiatric disorders. Prog. Neuropsychopharmacol. Biol. Psychiatry 70, 162–169. 10.1016/j.pnpbp.2016.01.013 26827636

[B36] Kim J. T. Shin D. K. (1978). A study based on the standardization of the STAI for Korea. Recent Med. 2, 69–75.

[B37] Kim S. J. Kim J. H. Youn S. C. (2010). The validation for Korean version of ruminative response scale. Korean J. Clin. Psychol. 29, 1–19. 10.15842/kjcp.2010.29.1.001

[B38] Kim S. J. Kwon J. H. Yang E. J. Kim J. H. Yu B. H. (2013). Confirmatory factor analysis of Korean-Ruminative Response Scale(K-RRS) in patients with depressive disorders. Cogn. Behav. Ther. Korea 13, 133–147.

[B39] Kline R. B. (2011). Principles and Practice of Structural Equation Modeling 3rd Edn. New York, NY: Guilford.

[B40] Klinitzke G. Romppel M. Hauser W. Brahler E. Glaesmer H. (2012). [The German version of the Childhood Trauma Questionnaire (CTQ): psychometric characteristics in a representative sample of the general population]. Psychother. Psychosom. Med. Psychol. 62, 47–51. 10.1055/s-0031-1295495 22203470

[B41] Leach L. S. Christensen H. Mackinnon A. J. Windsor T. D. Butterworth P. (2008). Gender differences in depression and anxiety across the adult lifespan: the role of psychosocial mediators. Soc. Psychiatry Psychiatr. Epidemiol. 43, 983–998. 10.1007/s00127-008-0388-z 18575787

[B42] Lok A. Bockting C. L. Koeter M. W. Snieder H. Assies J. Mocking R. J.. (2013). Interaction between the MTHFR C677T polymorphism and traumatic childhood events predicts depression. Transl. Psychiatry 3:e288. 10.1038/tp.2013.60 23900311 PMC3731792

[B43] MacCallum R. C. Browne M. W. Sugawara H. M. (1996). Power analysis and determination of sample size for covariance structure modeling. Psychol. Methods 1, 130–149. 10.1037/1082-989X.1.2.130

[B44] Maj M. Sartorius N. (2002). Depressive Disorder. Chichester: John Wiley & Sons Ltd.

[B45] Marcus D. K. Hughes K. T. Arnau R. C. (2008). Health anxiety, rumination, and negative affect: a mediational analysis. J. Psychosom. Res. 64, 495–501. 10.1016/j.jpsychores.2008.02.004 18440402

[B46] Mardia K. V. (1970). Measures of multivariate skewness and kurtosis with applications. Biometrika 57, 519–530. 10.1093/biomet/57.3.519

[B47] Marwaha S. Gordon-Smith K. Broome M. Briley P. M. Perry A. Forty L.. (2016). Affective instability, childhood trauma and major affective disorders. J. Affect. Disord. 190, 764–771. 10.1016/j.jad.2015.11.024 26615365

[B48] Nanni V. Uher R. Danese A. (2012). Childhood maltreatment predicts unfavorable course of illness and treatment outcome in depression: a meta-analysis. Am. J. Psychiatry 169, 141–151. 10.1176/appi.ajp.2011.11020335 22420036

[B49] Nolen-Hoeksema S. (1991). Responses to depression and their effects on the duration of depressive episodes. J. Abnorm. Psychol. 100, 569–582. 10.1037/0021-843X.100.4.569 1757671

[B50] Nolen-Hoeksema S. (2000). The role of rumination in depressive disorders and mixed anxiety/depressive symptoms. J. Abnorm. Psychol. 109, 504–511. 10.1037/0021-843X.109.3.504 11016119

[B51] Nolen-Hoeksema S. (2004). The Response Styles Theory. Chichester: John Wiley & Sons.

[B52] Nolen-Hoeksema S. (2012). Emotion regulation and psychopathology: the role of gender. Annu. Rev. Clin. Psychol. 8, 161–187. 10.1146/annurev-clinpsy-032511-143109 22035243

[B53] Nolen-Hoeksema S. Larson J. Grayson C. (1999). Explaining the gender difference in depressive symptoms. J. Pers. Soc. Psychol. 77, 1061–1072. 10.1037/0022-3514.77.5.1061 10573880

[B54] Nolen-Hoeksema S. Wisco B. E. Lyubomirsky S. (2008). Rethinking Rumination. Perspect. Psychol. Sci. 3, 400–424. 10.1111/j.1745-6924.2008.00088.x 26158958

[B55] Noto M. N. Noto C. Caribe A. C. Miranda-Scippa A. Nunes S. O. Chaves A. C.. (2015). Clinical characteristics and influence of childhood trauma on the prodrome of bipolar disorder. Rev. Bras. Psiquiatr. 37, 280–288. 10.1590/1516-4446-2014-1641 26692427

[B56] Paivio S. C. (2001). Stability of retrospective self-reports of child abuse and neglect before and after therapy for child abuse issues. Child Abuse Negl. 25, 1053–1068. 10.1016/S0145-2134(01)00256-3 11601597

[B57] Pennebaker J. W. Susman J. R. (1988). Disclosure of traumas and psychosomatic processes. Soc. Sci. Med. 26, 327–332. 10.1016/0277-9536(88)90397-8 3279521

[B58] Preacher K. J. Hayes A. F. (2004). SPSS and SAS procedures for estimating indirect effects in simple mediation models. Behav. Res. Methods Instrum. Comput. 36, 717–731. 10.3758/BF03206553 15641418

[B59] Raes F. Hermans D. (2008). On the mediating role of subtypes of rumination in the relationship between childhood emotional abuse and depressed mood: brooding versus reflection, *Depress*. Anxiety 25, 1067–1070. 10.1002/da.20447 18839403

[B60] Raes F. Hermans D. Williams J. M. Beyers W. Brunfaut E. Eelen P. (2006). Reduced autobiographical memory specificity and rumination in predicting the course of depression. J. Abnorm. Psychol. 115, 699–704. 10.1037/0021-843X.115.4.699 17100527

[B61] Rhee M. K. Lee Y. H. Park S. H. Sohn C. H. Chung Y. C. Hong S. K. (1995). A standardization study of beck depression inventory I; Korean version (K-BDI): reliability and factor analysis. Korean J. Psychopathol. 4, 77–95.

[B62] Sachs-Ericsson N. Verona E. Joiner T. Preacher K. J. (2006). Parental verbal abuse and the mediating role of self-criticism in adult internalizing disorders. J. Affect. Disord. 93, 71–78. 10.1016/j.jad.2006.02.014 16546265

[B63] Scher C. D. Stein M. B. Asmundson G. J. McCreary D. R. Forde D. R. (2001). The childhood trauma questionnaire in a community sample: psychometric properties and normative data. J. Trauma. Stress 14, 843–857. 10.1023/A:1013058625719 11776429

[B64] Sergi M. J. Rassovsky Y. Nuechterlein K. H. Green M. F. (2006). Social perception as a mediator of the influence of early visual processing on functional status in schizophrenia. Am. J. Psychiatry 163, 448–454. 10.1176/appi.ajp.163.3.448 16513866

[B65] Sobel M. E. (1982). Asymptotic confidence intervals for indirect effects in structural equation models. Sociol. Methodol. 13, 290–312. 10.2307/270723

[B66] Song Y. M. Lee H. K. Kim J. W. Lee K. (2012). Reliability and validity of the Korean version of beck depression inventory-II via the Internet: results from a university student sample. J. Korean Neuropsychiatr. Assoc. 51, 402–408. 10.4306/jknpa.2012.51.6.402

[B67] Spasojevic J. Alloy L. B. (2001). Rumination as a common mechanism relating depressive risk factors to depression. Emotion 1, 25–37. 10.1037/1528-3542.1.1.25 12894809

[B68] Spielberger C. D. (1983). State Trait Anxiety Inventory. Palo Alto, CA: Consulting Psychologists Press.

[B69] Villano C. L. Cleland C. Rosenblum A. Fong C. Nuttbrock L. Marthol M.. (2004). Psychometric utility of the childhood trauma questionnaire with female street-based sex workers. J. Trauma Dissociation 5, 33–41. 10.1300/J229v05n03_03 16957783 PMC1560176

[B70] Yuan K.-H. Bentler P. M. (1999). Structural Equation Modeling with Robust Covariances Los Angeles, CA: University of California.

[B71] Zahn R. Lythe K. E. Gethin J. A. Green S. Deakin J. F. Young A. H.. (2015). The role of self-blame and worthlessness in the psychopathology of major depressive disorder. J. Affect. Disord. 186, 337–341. 10.1016/j.jad.2015.08.001 26277271 PMC4573463

